# Impact of parasite genomic dynamics on the sensitivity of *Plasmodium falciparum* isolates to piperaquine and other antimalarial drugs

**DOI:** 10.1186/s12916-022-02652-2

**Published:** 2022-11-18

**Authors:** Dancan M. Wakoli, Bartholomew N. Ondigo, Douglas O. Ochora, Joseph G. Amwoma, Winnie Okore, Edwin W. Mwakio, Gladys Chemwor, Jackeline Juma, Raphael Okoth, Charles Okudo, Redemptah Yeda, Benjamin H. Opot, Agnes C. Cheruiyot, Dennis Juma, Amanda Roth, Benhards R. Ogutu, Daniel Boudreaux, Ben Andagalu, Hoseah M. Akala

**Affiliations:** 1 Department of Biochemistry and Molecular Biology, Egerton University, Egerton-Njoro, Kenya; 2 Department of Emerging and Infectious Diseases (DEID), United States Army Medical Research Directorate-Africa (USAMRD-A), Kenya Medical Research Institute (KEMRI)/ Walter Reed Project, Kisumu, Kenya; 3 Laboratory of Malaria Immunology and Vaccinology, National Institute of Allergy and Infectious Diseases, NIH, Bethesda, MD USA; 4 Department of Plant Sciences, Microbiology & Biotechnology, College of Natural Sciences, Makerere University, Kampala, Uganda; 5 Department of Biological Sciences, University of Embu, Embu, Kenya; 6 Department of Biomedical Sciences and Technology, School of Public Health and Community Development, Maseno University, Maseno, Kenya

**Keywords:** Artemisinin combined therapy, Antimalarial, Drug resistance, Sensitivity, Genomic, *Plasmodium falciparum*

## Abstract

**Background:**

Dihydroartemisinin-piperaquine (DHA-PPQ) is an alternative first-line antimalarial to artemether-lumefantrine in Kenya. However, recent reports on the emergence of PPQ resistance in Southeast Asia threaten its continued use in Kenya and Africa. In line with the policy on continued deployment of DHA-PPQ, it is imperative to monitor the susceptibility of Kenyan parasites to PPQ and other antimalarials.

**Methods:**

Parasite isolates collected between 2008 and 2021 from individuals with naturally acquired *P. falciparum* infections presenting with uncomplicated malaria were tested for in vitro susceptibility to piperaquine, dihydroartemisinin, lumefantrine, artemether, and chloroquine using the malaria SYBR Green I method. A subset of the 2019–2021 samples was further tested for ex vivo susceptibility to PPQ using piperaquine survival assay (PSA). Each isolate was also characterized for mutations associated with antimalarial resistance in *Pfcrt*, *Pfmdr1*, *Pfpm2/3*, *Pfdhfr*, and *Pfdhps* genes using real-time PCR and Agena MassARRAY platform. Associations between phenotype and genotype were also determined.

**Results:**

The PPQ median IC_50_ interquartile range (IQR) remained stable during the study period, 32.70 nM (IQR 20.2–45.6) in 2008 and 27.30 nM (IQR 6.9–52.8) in 2021 (*P*=0.1615). The median ex vivo piperaquine survival rate (IQR) was 0% (0–5.27) at 95% CI. Five isolates had a PSA survival rate of ≥10%, consistent with the range of PPQ-resistant parasites, though they lacked polymorphisms in *Pfmdr1* and *Plasmepsin* genes. Lumefantrine and artemether median IC_50_s rose significantly to 62.40 nM (IQR 26.9–100.8) (*P* = 0.0201); 7.00 nM (IQR 2.4–13.4) (*P* = 0.0021) in 2021 from 26.30 nM (IQR 5.1–64.3); and 2.70 nM (IQR 1.3–10.4) in 2008, respectively. Conversely, chloroquine median IC_50_s decreased significantly to 10.30 nM (IQR 7.2–20.9) in 2021 from 15.30 nM (IQR 7.6–30.4) in 2008, coinciding with a decline in the prevalence of *Pfcrt* 76T allele over time (*P* = 0.0357). The proportions of piperaquine-resistant markers including *Pfpm2/3* and *Pfmdr1* did not vary significantly. A significant association was observed between PPQ IC_50_ and *Pfcrt* K76T allele (*P*=0.0026).

**Conclusions:**

Circulating Kenyan parasites have remained sensitive to PPQ and other antimalarials, though the response to artemether (ART) and lumefantrine (LM) is declining. This study forms a baseline for continued surveillance of current antimalarials for timely detection of resistance.

**Supplementary Information:**

The online version contains supplementary material available at 10.1186/s12916-022-02652-2.

## Background

Malaria remains one of the fatal parasitic diseases causing over 200 million malaria cases and over 600,000 deaths annually. Sub-Saharan Africa (SSA) accounts for 95% of the disease burden, with most cases occurring in children below five years and pregnant women [1]. In Kenya, malaria is a major public health problem. Over 70% of its population is at risk, especially in the endemic lake region of western Kenya [2, 3], while it has been suggested that a decline in prevalence proffer increased risk of severe disease across all age groups [3]. This high burden is partly sustained by the rapid development of *P. falciparum* resistance to antimalarial drugs and other factors [1, 4], malaria remains preventable and treatable [5].

Antimalarial drugs, alongside the RTS,S vaccine, and vector control, are the preferred means of malaria control [1, 6]. The recent decrease in the global malaria burden has been attributed partly to the deployment of artemisinin combination therapy (ACTs) and other interventions [6–9]. Recommended ACTs include; artemether-lumefantrine (AL), dihydroartemisinin-piperaquine (DHA-PPQ), artesunate-amodiaquine (ART-AQ), artesunate plus sulphadoxine-pyrimethamine (AS-SP), artesunate-mefloquine (AS-MQ) and artesunate-pyronaridine (AS-PY) [1, 10]. Though ACTs have had marked success in the last two decades [10], the recent emergence of artemisinin resistance in Southeast Asia, and emerging signals of the same from Rwanda and Northern Uganda is a threat to the continued use of ACTs in SSA [11–13].

Dihydroartemisinin-piperaquine combination is the recommended alternative first-line drug to artemether-lumefantrine (AL) and Artesunate-Amodiaquine in SSA [7]. This treatment was adopted by the Kenya Ministry of health in 2009 [2, 14]. PPQ confers a 60-day prophylactic effect after treatment [7, 13–15], and has never been used as a monotherapy in Africa [16]. Recent studies show the emergence, development, and spread of DHA-PPQ resistance in Cambodia, Vietnam, and Thailand [10, 17, 18]. The emergence of resistance warrants monitoring of the efficacy of DHA-PPQ in SSA [7, 19, 20] since historical patterns of antimalarial drug resistance patterns show that resistance emerging from any part of the world often reaches Africa [21].

Resistance to DHA-PPQ has been attributed to drug pressure, a longer half-life of PPQ [17]. Studies suggest that resistance to PPQ is caused by genetic mutations within the *Plasmepsin-2/3* (*Pfpm2/3)* complex, *Pfmdr1*, and non-synonymous SNPs in *Pfcrt*, *PfK13*, and *Pfexo* genes [6, 14, 19, 22, 23]. There is a scarcity of genotypic and phenotypic data that can inform on piperaquine resistance in Kenya currently as the country continues to deploy DHA-PPQ alternative treatment. To fill this gap, susceptibility of Kenyan isolates to PPQ and other selected antimalarial drugs was assessed using growth inhibition assays and genomic analyses. Findings from this study underscore parasite susceptibility to antimalarials including DHA-PPQ before the widespread use of DHA-PPQ. The findings will form a baseline to support tracking how natural infections will respond to DHA-PPQ as the use of this treatment becomes more widespread.

## Methods

### Study design, ethical statement, and subjects

This study was approved by the Kenya Medical Research Institute (KEMRI) Scientific and Ethics Review Unit and the Walter Reed Army Institute of Research (WRAIR) institutional review boards. Archived (2008–2017) and freshly collected (2018–2021) samples were collected under protocol numbers: KEMRI #1330 and #3628, WRAIR #1384, and #2454.

**Fig. 1:**
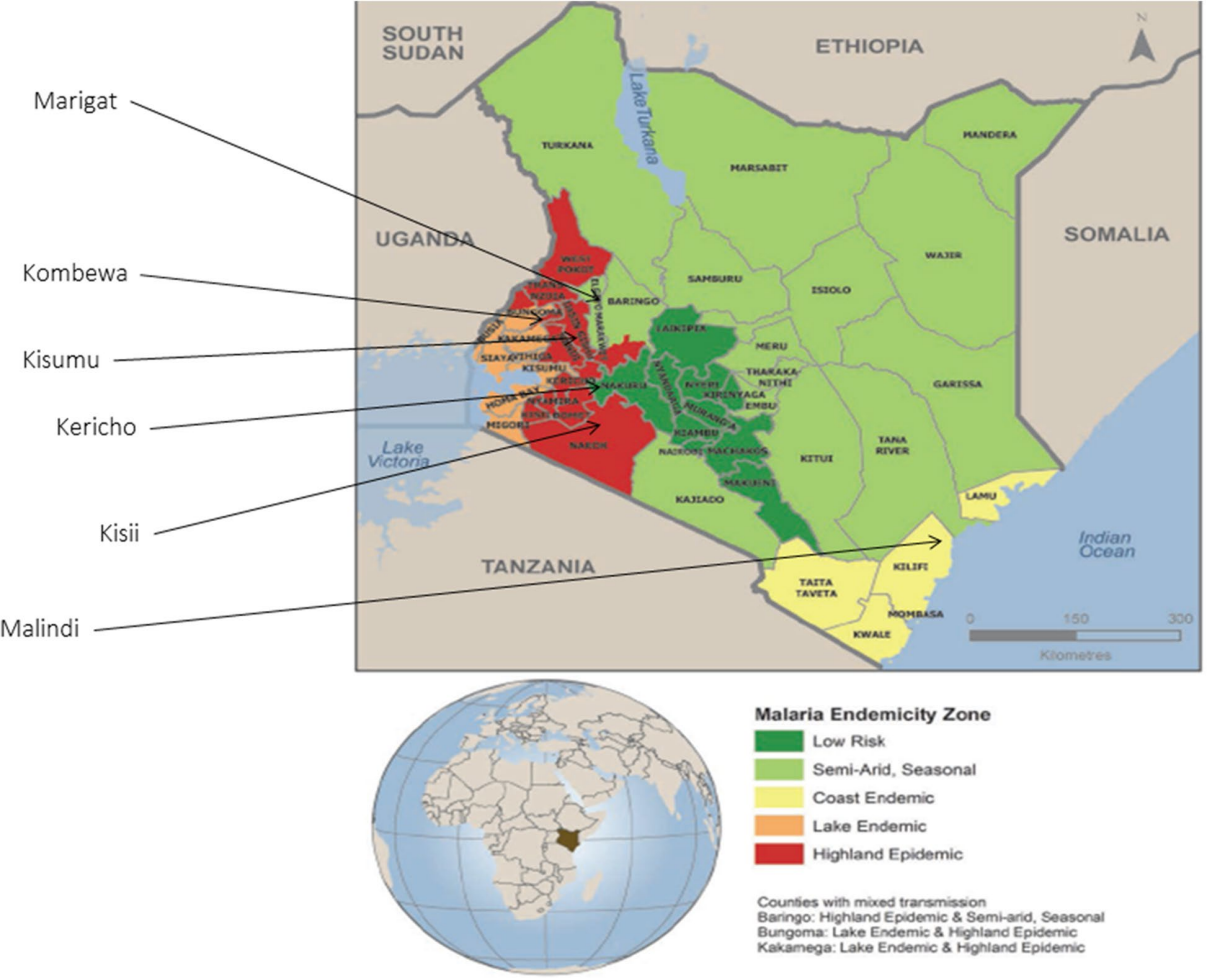
Map of Kenya depicting the six hospital surveillance sites

The samples were collected from Kenya Ministry of Health (MOH) sentinel hospital sites located in four of Kenya’s five different malaria ecological zones, namely; the lake endemic region (Kisumu East County and Kisumu West/Kombewa sub-county hospitals), Coastal endemic region (Malindi sub-county hospital), highland epidemic-prone areas (Kericho and Kisii county referral hospitals), and semi-arid/seasonal prone area (Marigat sub-county hospital) (Fig. 1) [2].

Individuals aged ≥ 6 months, presenting at the health care facilities outpatient department with symptoms consistent with uncomplicated malaria and confirmed positive for *Plasmodium falciparum* by malaria rapid diagnostic test (mRDT) (Parascreen Pan/Pf; Zephyr Biomedicals, Verna, Goa, India) or microscopy were enrolled into the study upon obtaining a written informed consent. Written informed assent for individuals under 18 years was obtained in accordance with the laws of the government of Kenya. Individuals with diminished autonomy such as prisoners, individuals previously enrolled in the study during the same calendar year, infants weighing < 5 kg, and those individuals whom the opinion of the attending medical officer considered that they could be adversely affected by the drawing of 2.5 mL of blood were excluded. About 2–3 mL of venous blood was obtained from those enrolled before treatment. Subsequently, treatment for these individuals was based on the Kenya Ministry of Health guidelines on case management of uncomplicated malaria, as reported in our previous study [24].

### Sample collection and preparation

The venous blood sample collected from each individual was distributed in different tubes as follows, 0.5 mL in acid citrate dextrose (ACD) tube (Becton-Dickinson, Franklin Lakes, NJ, USA) for immediate ex vivo growth inhibition assay and culture adaptation, 0.5 mL in sodium heparin tube (Becton-Dickinson, Franklin Lakes, NJ, USA) for in vitro studies, 1 mL in EDTA tube (Becton-Dickinson, Franklin Lakes, NJ, USA) for leukocyte depletion and genomic analysis, two drops on each of the two glass slides for microscopic parasite examination, and three blood spots of each 100 μL on FTA filter paper (Whatman Inc., Bound Brook, NJ) for DNA extraction and genomic analysis [23, 24]. Collected samples were transported to the central laboratory for processing, culture adaptation, genomic, growth inhibition assays, and storage, as described earlier [25].

### Reference *Plasmodium falciparum* strains

The following reagents were obtained through BEI Resources, NIAID-NIH: *Plasmodium falciparum*, strain 3D7, and MRA-102 were contributed by Daniel J. Carucci; Strain D6, MRA-285, and Strain W2, MRA-157, contributed by Dennis E. Kyle.

### Reference antimalarial drugs

The reference antimalarials drugs piperaquine (PPQ), dihydroartemisinin (DHA), lumefantrine (LM), artemether (ART), and chloroquine (CQ) were donated by the WorldWide Antimalarial Resistance Network (WWARN) External Quality Assurance Programme, Bangkok, Thailand [26].

### In vitro parasite adaptation

Archived samples comprising previously collected field isolates and laboratory reference strains (3D7/D6 [sensitive to chloroquine and resistant to mefloquine] and W2 [chloroquine resistant and sensitive to mefloquine]) were pulled from the liquid nitrogen, thawed, and maintained in continuous culture in RPMI plus supplements consisting of 10.4 g RPMI 1640 medium [Gibco BRL, UK], 25 mM HEPES, 5.5 mM D-glucose, 10 mM hypoxanthine, 7.5% sodium bicarbonate, 15% (vol/vol) heat-inactivated pooled ABO human sera and 0.1 μl/mL; gentamycin per litre of distilled water, adjusted at 2% hematocrit with uninfected human red blood cells and maintained at 37°C, 5% CO_2_, 5% O_2_, 90% N_2_, and humidified environment as described [17, 25, 27, 28]. The cultures were monitored at 24 hourly intervals till attaining parasitemia of 3–8%. Cultures that could not attain this parasitemia were considered unsuccessful [17, 29].

### Drug sensitivity testing by SYBR Green I-based assay

SYBR Green I-based in vitro and immediate ex vivo drug sensitivity assay was used to test each *P. falciparum* field isolate and control against a panel of five antimalarial drugs, namely, piperaquine (PPQ), dihydroartemisinin (DHA), lumefantrine (LM), artemether (ART), and chloroquine diphosphate (CQ) [25]. Drugs were prepared to the desired concentration as described earlier [25]. Freshly collected samples were subjected to immediate ex vivo assay [29, 30] while culture-adapted field isolates and reference clones that had attained 3–8% parasitemia in continuous culture were subjected to in vitro malaria SYBR Green I assay [25, 31]. Briefly, the sample parasitemia was adjusted to 1% at 2% hematocrit using complete media and uninfected O+ RBCs [25, 30] and mixed to homogeneity. 100 μl of this mixture was loaded to each well of the 96-well microtiter plates (catalog no: 167008 Nunc, Inc, Roskilde, Denmark) containing different concentrations of drug aliquots, incubated at 37°C under a 90% N_2_, 5% O_2_, and 5% CO_2_ humidified environment and terminated 72 h later [25, 31]. Lysis buffer containing SYBR Green I dye was added and then incubated for 24 h in the dark. Readings were then done using Tecan Genios Plus® (Tecan US, Inc., Durham, NC) which gave the relative fluorescence units (RFUs). Using these readouts, the strength of inhibition of each drug, and the 50% inhibition concentration (IC_50_) were calculated using Graphpad prism® 8.1 for Windows® software (Graphpad Software, San Diego, CA, USA) using non-linear regression analysis of the dose-response curve [25, 31].

### Piperaquine survival assay (PSA)

Fresh samples collected from malaria patients presenting themselves at Kisumu West and Kisumu East hospital sites between 2019 and 2021 were analyzed using the immediate ex vivo PSA as described by Duru and coworkers [17]. Samples at equal or greater than 1% parasitemia, arriving at the central laboratory within 6 h after phlebotomy at ambient temperature, were reconstituted to 1% parasitemia, and 2% hematocrit, and then subjected to the assay. Similarly, the samples maintained in continuous culture were synchronized as described initially [30] to attain 0–3 h old rings prior to initiation of PSA [17]. Importantly, a blood smear was made from the reconstituted samples at 1% parasitemia for reference as initial parasitemia. Aliquot of 900 μl of the reconstitutes parasitized red blood cell sample were loaded to a 48-well culture plate (Nunc, Inc., Roskilde, Denmark) containing 100 μl of 2000 nM piperaquine tetraphosphate tetrahydrate giving a final concentration of 200 nM piperaquine (exposed culture) and 89.5% dimethyl sulfoxide (Sigma-Aldrich, ST. Louis, Germany) (non-exposed culture/control) in triplicates [17]. The assay plates were incubated for 48 h at 37°C in a mixture of 5% O_2_, 5% CO_2_, and 90% N_2_ in a humidified environment as earlier described [17]. After 48 h incubation, contents of the assay wells were separately washed three times in RPMI, then resuspended in a drug-free RPMI plus supplements medium for a further 24 h [17]. Thin blood smears were prepared, methanol-fixed, and stained by 10% Giemsa (Sigma-Aldrich, ST. Louis, Germany) for 20 min [17]. The percentage of viable *P. falciparum* which developed into second-generation rings or trophozoites in the exposed and non-exposed cultures was determined by assessing parasites with normal morphology in 10,000 red blood cells by two independent microscopists blinded to the clinical data [17]. Piperaquine (PPQ) sensitivity was expressed as median survival rate and computed as earlier described [17]. Clinical isolates with a piperaquine survival rate ≥10% were considered PPQ-resistant and vice versa. The assay was considered valid when the growth rate (parasite in the control at 72 h {non-exposed})/initial parasitemia before testing at 72 h) was ≥ 1 for ex vivo.

### Analysis of *Pfmdr1*, *Pfpm2*, and *Pfpm3* copy number variation

Parasite nucleic acids were extracted from whole blood or dry blood spots (DBS) using QIAamp® DNA mini kit (Qiagen, Inc., Germany) following the manufacturer's instructions. Extracted DNA was stored at -20°C. *Plasmepsin*-*2* (*Pfpm2*), *Plasmepsin*-*3* (*Pfpm3*), and *P. falciparum* multi-drug-resistant transporter 1 (*Pfmdr1*) primers for real-time PCR (Applied Biosystems Inc., Foster City, CA) method were designed using an online GeneScript tool as described earlier [23] and purchased from applied biosystems (Foster City, CA). Copy number variation in the genes was analyzed as described by Ansbro and coworkers with some modifications [23]. Briefly, each 20 μl PCR reaction composed of 10 μl of Taqman multiplex mastermix, 1 μl of forward and reverse primer for each gene and *β-tubulin*, 1 μl of each probe, 2 μl nuclease-free water, and 2 μl of template DNA. In each real-time PCR run, 3D7 and DD2 strains of *P. falciparum* were used as controls with one and multiple copies of *Pfmdr1*, respectively; nuclease-free water was also included as a negative control [23, 32]. A housekeeping gene, *β-tubulin*, and 3D7 were used as internal control and calibrator respectively for the assay [23, 26, 32]. Test samples were assayed in triplicates for real-time PCR [33]. The cycling conditions were; the initial holding step of 95°C for 5 min followed by 40 cycles of 95°C for 15 s and 60°C for 30 s [34]. PCR cycle threshold (CT) values were generated for all the DNA isolates. Only those with CT value ≤ 32 were considered for analysis by SDS software (version 2.0.6; Applied Biosystems Inc., Foster City, CA). The relative quantification method, 2^-ΔΔCt^ was used to estimate copy number variation for each test gene [25, 30]. Field isolates with copy number ≥1.5 were interpreted as true multiple copies [35].

### Genotyping of piperaquine resistance genes on the MassARRAY platform

A matrix-assisted laser desorption ionization-time of flight mass spectrometry (MALDI-TOF MS) coupled with a single-base extension PCR (iPLEX PCR) (Agena Biosciences, San Diego, CA) was used to analyze single nucleotide polymorphisms (SNPs) in *Pfdhps, Pfdhfr, Pfcrt* and *Pfmdr1* genes of *P. falciparum* following the manufacturer’s protocol. Field isolates from all study sites collected between 2008–2013 and 2018–2021 study periods were analyzed. The primers for this assay were designed by the Agena Bioscience Assay Design Suite (ADS) version 2.0 (Agena Bioscience, San Diego, CA). The primary PCR reaction was run on a GeneAmp 9700 PCR system (Applied Biosystems, Foaster City, California, USA), using the Agena Bioscience PCR Reagent Set. Each reaction mixture comprised of 0.5 μL 10x PCR buffer, 1.8 μL HPLC-grade water, 0.40 μL MgCl_2_, 0.10 μL dNTPs mix, 1.00 μL primer mix, 0.20 μl Taq polymerase enzyme, and 1 μL of DNA. This PCR was set at the following conditions; initial denaturation at 95°C for 2 min, 44 cycles of 95°C for 30 s, 56°C for 30 S, and 72°C for 60 S, and a final extension at 72°C for 5 min. The unincorporated dNTPs from the primary PCR were dephosphorylated by the shrimp alkaline phosphatase enzyme (SAP). The iPLEX PCR was then performed on a GeneAmp 9700 PCR system with the iPLEX Gold Reaction Kit (Agena Bioscience), following the manufacturer's instructions. For PCR, 2 μL of the prepared extension primer cocktail was added to each well. Cycling conditions for this step were as follows; initial denaturation at 94°C for 30 S, 40 cycles of one step at 94°C for 5 S with five sub-cycles of 52°C for 5 S, and 80°C for 5 S, and final extension at 72°C for 3 min. Extended products from the later step were conditioned using a resin (Agena Bioscience) and HPLC-grade water. Approximately 10 nL of the extended products was dispensed into a 96-well SpecroCHIP (Agena Bioscience) using the MassARRAY Nano-dispenser RS1000 (Agena Bioscience) followed by automatic data acquisition on the mass spectrometer using SpectroAcquire software.

### Data analysis

In vitro susceptibility and genotype alongside ex vivo PSA data were expressed as median IC_50_s with interquartile range (IQR) and proportions, respectively. The isolates were grouped into three periods, namely, 2008–2013, 2014–2017, and 2018–2021. Differences in IC_50_s between study periods and study sites were quantified using the Kruskal-Wallis (*H*-test). Post hoc analyses were done using Dunn's multiple comparison tests. Proportions were examined using the Chi-square test (*χ*^2^) and Fisher exact test. Associations between genotype and drug susceptibilities (IC_50_s) were calculated using the Kruskal-Wallis *H*-test and Mann-Whitney *U* test respectively. The drug in vitro activity correlations were calculated using the Spearman correlation coefficient. All statistical tests were carried out in GraphPad version 8.0 (GraphPad Software, Inc., San Diego, CA, USA). Two-sided *P* value < 0.05 was considered statistically significant.

## Results

### Analysis of *P. falciparum* in vitro susceptibility to antimalarial drugs over time and by Hospital site

**Fig. 2:**
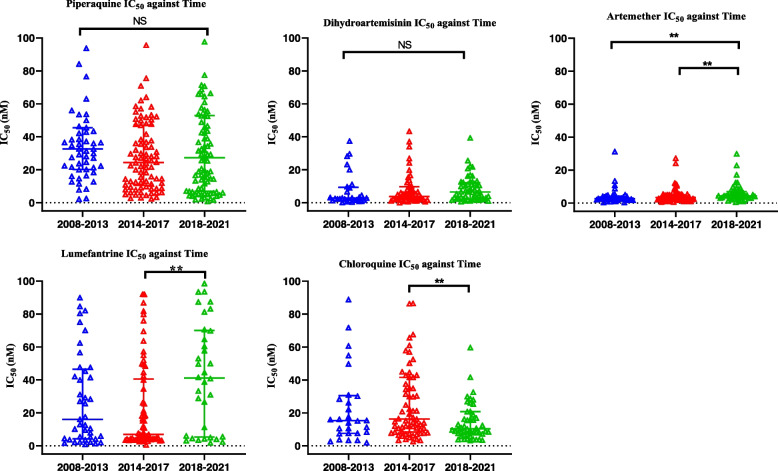
Temporal changes of parasite’s response to antimalarials between 2008–2013, 2014–2017, and 2018–2021. Scatter plots with bars and whiskers showing median IC_50_s and interquartile range (nM). *P* < 0.05 representative of statistically significant change in IC_50_ represented by ** for chloroquine, lumefantrine, and artemether while the rest remained unchanged

A total of 239/252 clinical field isolates were collected between 2008 and 2021 (Additional file 1: Table S1) and successfully assayed for drug susceptibility against PPQ, DHA, LM, ART, and CQ using the standard malaria SYBR Green I method. Chloroquine-sensitive (3D7/D6) and resistant (W2) reference clones were analyzed in parallel as assay controls (Additional file 2: Fig. S1). Assessment of the field isolates against lumefantrine and artemether showed a significant increase in median IC_50_ across the study periods (Fig. 2 and Additional file 1: Table S2). Conversely, there was a significant decrease in chloroquine median IC_50_ across the study time points (Fig. 2 and Additional file 1:Table S2). Significant temporal changes in susceptibility of the parasites to LM and CQ were observed between 2014–2017 and 2018–2021 study periods, whereas for ART, variation in susceptibility was shown between 2008–2013 and 2018–2021 alongside 2014–2017 and 2018–2021 study periods (Fig. 2). However, PPQ and DHA median IC_50_ remained unchanged during the study period between 2008 and 2021 (Additional file 1: Table S2).

**Fig. 3:**
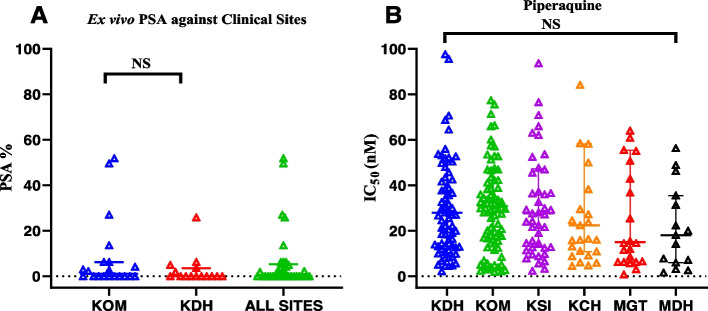
Scatter plot showing **A** ex vivo piperaquine survival rates of Kenyan clinical isolates from two locations and **B** susceptibility of parasites from six different geographic locations in Kenya to piperaquine using malaria SYBR green I assay. Horizontal bars and whiskers represent the median and interquartile range of susceptibility

Additionally, the median fifty percent inhibitory concentration (IC_50_) of piperaquine against clinical isolates was 32.62 nM (20.1–45.6) for 2008–2013, 27.64 nM (12.4–47.6) for 2014–2017 and 27.32 nM (6.9–52.9) for 2018–2021 (Additional file 1: Table S2) showing a marginal decline in IC_50_ between 2008 and 2021, although this was not statistically significant (*P* = 0.1615) (Fig. 2 and Additional file 1: Table S2). Samples from Malindi Sub-County hospital had significantly lower IC_50_s than those from Kisumu East County hospital at median IC_50_s and IQRs of; 37.16 nM (18.9–53.2) and 18.11 nM (6.1–35.5), respectively, (*P* = 0.0451) (Fig. 3 and Additional file 1: Table S3). We observed a significant decrease in the PPQ median IC_50_s between 2008–2013 and 2014–2017 study periods in field isolates collected from Kisii county hospital compared to other sites (*P* = 0.0485) (Additional file 1: Table S4).

### In vitro drug activity correlation between 2008 and 2021

A significant correlation was observed between the activity of two artemisinin derivatives; (DHA and ART) (rho=0.4, *P*=0.0003) (Table 1). Contrary, there was no correlation observed between the activity of LM (*P*=0.6097) or CQ (*P*= 0.8068) and artemisinin derivatives; DHA and ART (*P*= 0.4679 and *P*= 0.8230, respectively) (Table 1).

**Table 1:** Correlation between in vitro responses of Kenyan *P. falciparum* isolates to piperaquine and other antimalarials

Drug pair	Number of isolates	Correlation coefficient (***r***)	***P***-value
PPQ versus DHA	77	0.0840	0.4679
PPQ versus LM	80	−0.0579	0.6097
PPQ versus ART	80	0.0254	0.8230
PPQ versus CQ	80	0.0278	0.8068
DHA versus LM	77	0.2105	0.0662
DHA versus ART	77	0.4036	0.0003**
DHA versus CQ	77	0.0589	0.6111
LM versus ART	80	0.0602	0.5957
LM versus CQ	80	0.1328	0.2402
ART versus CQ	80	0.0092	0.9355

The correlation for each pair of drugs per isolate is based on IC_50_ values

*PPQ* piperaquine, *DHA* dihydroartemisinin, *LM* lumefantrine, *ART* artemether, *CQ* chloroquine

** *P* < 0.05 consistent with statistically significant difference assessed by Spearman correlation coefficient analyses

### *P. falciparum* susceptibility to piperaquine by Piperaquine Survival Assay (PSA)

A total of 34 freshly collected field isolates characterized by immediate ex vivo PSA had an overall piperaquine median survival rate and IQR of 0% (0–5.3). 29/34 (85%) and this was categorized as PSA rate < 10% therefore below resistance threshold, 14 of these were from Kisumu West sub-county hospital and 15 from Kisumu East County referral hospital while remaining five isolates had piperaquine survival rate ≥10% categorized as piperaquine resistant. Out of these five, four were from Kisumu West sub-county hospital, and one from Kisumu East County referral hospital. The median piperaquine survival rate for samples from the two hospital sites was comparable, Kisumu West Hospital 1.25% (0–6.2) versus Kisumu East Hospital, 0% (0–3.5) (Fig. 3). Genomic analyses of isolates with PSA rate ≥10 did not detect any piperaquine resistance candidate marker. The *Pfpm2*, *Pfpm3*, and *Pfmdr1* copy numbers did not differ among the isolates with PSA ≥ 10 versus PSA < 10 (*P* > 0.999). Additionally, the PPQ median IC_50_ 31.04 nM (31.5–34.3), of isolates that showed PSA survival rates ≥ 10% did not differ from the IC_50_s 38.86 nM (18.9–67.0) of those that showed PSA rates < 10 (*P* = 0.4833).

### Assessment of copy number variation for *Pfmdr1*, *Pfpm2*, and *Pfpm3* genes

A total of 280, 286, and 296 samples were successfully analyzed for copy number variation for the *Pfmdr1*, *Pfpm2*, and *Pfpm3* genes, respectively. The overall median copy number and IQR for all the genes were 1.02 (0.9–1.2) for *Pfmdr1*; 0.99 (0.9–1.1) for *Pfpm2*, and 1.1 (0.9–1.2) for *Pfpm3*. Using a copy number threshold of 1.5 to define multiple gene copy isolates [36], the frequencies of samples having multiple copies for *Pfmdr1* and *Pfpm2* were stable over the study period (1.7–3.2%) (Table 2 and Additional file 2: Fig. S2). In contrast, *Pfpm3* multiple copy number decreased marginally over time from 10.1% to 4% (*P* = 0.4522) (Table 2 and Additional file 2: Fig. S2). Temporal trends of copy number estimate across the study period revealed absence of statistically significant differences in the proportions of *P. falciparum* isolates with multiple copies of *Pfpm2* (*P*=0.8318), *Pfpm3* (*P* =0.4522), and *Pfmdr1* (*P* =0.7304) genes between the three study periods, namely, 2008–2013, 2014–2017 and 2018–2021 (Table 2). The distribution of these mutations in different hospital study sites is as shown (Additional file 1: Table S5).

**Table 2:** Frequency of Kenyan parasites harboring *Pfpm2/3* and *Pfmdr1* multiple copies between 2008 and 2021

Study period								
		2008–2013		2014–2017		2018–2021		***P-***value
Genotype		***N***	Proportion	***N***	Proportion	***N***	Proportion	
***Pfmdr1***	Single copy	57	98.3%	122	96.1%	92	96.8%	
	Multi-copy	1	1.7%	5	3.9%	3	3.2%	0.7304
***Pfpm2***	Single copy	65	97.0%	118	98.3%	97	98.0%	
	Mult-copy	2	3.0%	2	1.7%	2	2.0%	0.4522
***Pfpm3***	Single copy	80	89.9%	100	93.5%	96	96.0%	
	Multi-copy	9	10.1%	7	6.5%	4	4.0%	0.8318

*Pfmdr1 Plasmodium falciparum* multi-drug resistance 1 gene, *Pfpm2 Plasmodium falciparum plasmepsin* 2, *Pfpm3 Plasmodium falciparum plasmepsin 3*, *N* number of samples

### Prevalence of *P. falciparum* genetic polymorphisms associated with antimalarial resistance

Analysis of *P. falciparum* isolates for genetic polymorphisms that confer antimalarial resistance revealed a significant decrease in frequencies of *Pfcrt* K76T (*P* = 0.0357) and *Pfdhps* 437G (*P* = 0.0218) mutants from 39.3% (11/28) to 0% (0/9) and 50% (8/16) to 0% (0/9), respectively between the study periods, 2008–2013 and 2018–2021. Conversely, *Pfdhps* 436F/A *(P* = 0.0227) mutant allele proportion increased significantly from 0% (0/15) to 75% (3/4) over time (Table 3). *Pfmdr1* 86Y allele proportions dropped from 25.7% (9/35) to 0% (0/9) across the study period but this was not statistically significant (*P* = 0.1674). On the contrary, no evidence of significant changes between the study periods was observed for other polymorphisms (Table 3).

**Table 3:** Frequency of antimalarial resistance polymorphisms in *P. falciparum* isolates collected from Kenya between 2008 and 2021

Polymorphism	2008–2013	2018–2021	***P***-value
	*n* (%)	*n* (%)	
*Pfmdr1* N86Y	9 (25.7)	0 (0)	0.1674
*Pfmdr1* Y184F	16 (43.2)	3 (42.9)	> 0.999
*Pfcrt* K76T	11 (39.3)	0 (0)	0.0357**
*Pfdhps* S436F/A	0 (0)	3 (75)	0.0227**
*Pfdhps* A437G	8 (50)	0 (0)	0.0218**
*Pfdhps* A581G	0 (0)	0 (0)	NA
*Pfdhps* A613S	0 (0)	0 (0)	NA
*Pfdhfr* C59R	18 (81.8)	13 (100)	0.2735
*Pfdhfr* I164L	2 (9.5)	0 (0)	0.5343

*Pfmdr1 Plasmodium falciparum* multi-drug resistance 1 gene, *Pfcrt Plasmodium falciparum* chloroquine resistance transporter gene, *Pfdhps Plasmodium falciparum* dihydropteroate synthetase, *Pfdhfr Plasmodium falciparum* dihydrofolate reductase, *NA* not applicable

** *P* < 0.05 consistent with statistically significant differences in frequency of polymorphisms across the period by Fisher exact test

### Association between in vitro piperaquine susceptibility and PPQ resistance molecular markers

**Fig. 4:**
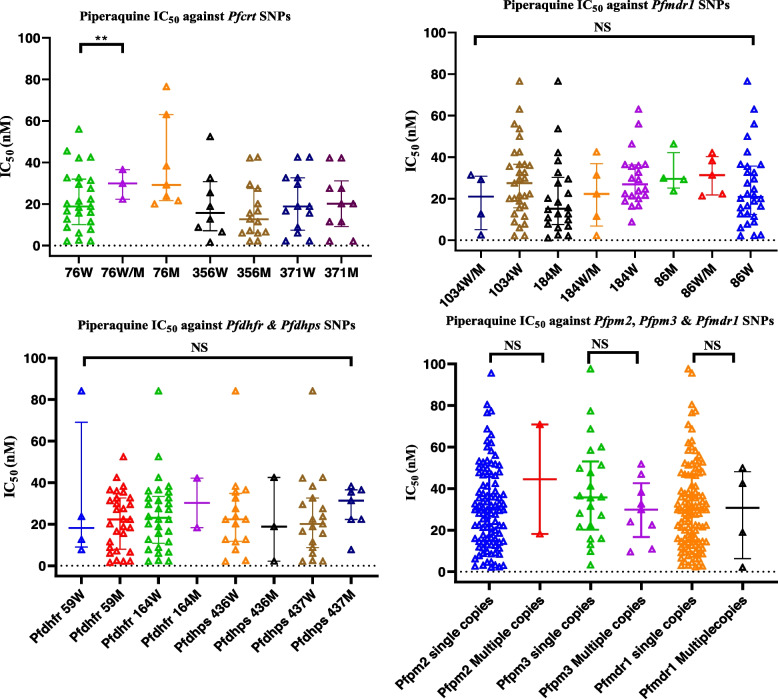
Association between drug resistance polymorphisms and in vitro susceptibility to piperaquine measured by SYBR green I assay. Scatter plots with horizontal bars and whiskers representing median IC_50_s and interquartile ranges for each locus. M-mutant genotype, W- wild-type genotype, W/M- mixture of mutant and wild-type genotype in an infection. *Pfcrt, Plasmodium falciparum* chloroquine resistance gene; *Pfmdr1*, *Plasmodium falciparum* multidrug resistance gene 1; *Pfdhps*, *Plasmodium falciparum* dihydropteroate synthetase gene; *Pfdhfr*, *Plasmodium falciparum* dihydrofolate reductase gene; *Pfpm2*, *Plasmodium falciparum plasmepsin* 2 gene; and *Pfpm3*, *Plasmodium falciparum plasmepsin* 3 gene

Assessment of association between PPQ IC_50_ values and mutations in *Pfmdr1*, *Pfcrt*, *Pfdhfr*, *Pfdhps*, *Pfpm2/3*, and *Pfmdr1* copy number variation using Mann Whitney-*U* test and Kruskal Wallis H test showed a significant relationship between PPQ IC_50_s and, *Pfcrt* K76T SNP (*P* = 0.0026) (Table 4). Post-hoc analysis revealed that parasites harboring the *Pfcrt* K76T mixed species genotype had higher median in vitro PPQ IC_50_ 29.90 nM compared to the wild-type isolates 18.86 nM (*P* = 0.0026) (Fig. 4). However, there was no significant association between *Pfdhps*, *Pfdhfr*, *Pfmdr1* SNPs, *Pfpm2/3*, *Pfmdr1* multiple copies, and PPQ in vitro sensitivity (IC_50_) (Table 4, Fig. 4).

**Table 4:** Association between piperaquine (PPQ) resistance mutations (SNPs as well as copy number variation (CNV)) with in vitro PPQ IC_50_s of Kenyan field isolates collected between 2008 and 2021

In vitro SYBR Green I assay					
Gene Polymorphisms/Assay		*N*	Proportions (%)	PPQ median IC_50_ (IQR) nM	*P* value
***P. falciparum*** **multidrug resistance 1 gene,** ***Pfmdr1*** SNPs	N86	34	75.6	23.5 (12.7–44.4)	0.26
	86N/Y	6	13.3	34.9 (22.0–80.5)	
	86Y	5	11.1	29.9 (26.5–288.5)	
	Y184	22	47.8	29.5 (21.1–39.0)	0.55
	184Y/F	6	13.0	26.9 (9.2–80.7)	
	184F	18	39.1	23.9 (11.4–45.0)	
	A1034	34	85.0	29.7 (21.1–39.0)	0.81
	1034T	6	15.0	30.3 (10.3–279.4)	
***P. falciparum*** **chloroquine resistance transporter 1 gene,** ***Pfcrt*** SNPs	K76	25	65.8	18.9 (10.1–31.9)	0.0026^**^
	76K/T	5	13.2	36.6 (26.1–249.9)	
	76T	8	21.0	33.8 (22.2–73.2)	
	T356	9	37.5	18.9 (7.7–42.6)	0.73
	356C	15	62.5	15.7 (6.5–29.5)	
	G371	10	64.3	16.6 (8.0–30.3)	0.96
	371T	18	35.7	20.2 (9.2–31.2)	
***P. falciparum*** **dihydrofolate reductase gene,** ***Pfdhfr*** SNPs	C59	4	11.8	18.3 (7.8–69.1)	0.98
	59R	30	88.2	22.4 (1.7–33.4)	
	I164	30	92.3	23.2 (10.8–33.4)	0.58
***P. falciparum dihydropteroate synthetase*** **gene,** ***Pfdhps*** SNPs	164L	2	7.7	30.3 (18.3–42.2)	
	S436	16	84.2	22.4 (11.8–34.7)	0.88
	436F	3	15.8	18.9(2.2–416.1)	
	A437	19	73.1	20.2 (8.8–32.7)	0.33
	437G	7	26.9	31.4 (22.4–36.6)	
***P. falciparum*** **multidrug resistance 1 gene,** ***Pfmdr1*** CNV	Single copy	271	96.8	29.9 (14.4–47.4)	0.48
	Multiple copies	9	3.2	46.1 (12.1–99.9)	
***P. falciparum plasmepsin*** **2 gene,** ***Pfpm2*** CNV	Single copy	280	97.9	29.9 (14.4–47.2)	0.63
	Multiple copies	6	2.1	44.6 (18.3 –71.0)	
***P. falciparum plasmepsin*** **3 gene,** ***Pfpm3*** CNV	Single copy	276	92.9	30.0 (14.5–47.6)	0.72
	Multiple copies	20	7.1	31.3 (19.6–48.2)	

** Show *P* < 0.05 consistent with statistically significant differences in frequency over time using Kruskal-Wallis (*H*) tests for three groups and Mann-Whitney (*U*) test for two groups

## Discussion

This study showed that Kenyan *P. falciparum* field isolates are susceptible to PPQ using growth inhibition assays and molecular marker analyses. There was a low frequency of the PPQ resistance polymorphisms in *plasmepsin* 2/3 (*Pfpm2/3*) and *Pfmdr1* genes reported elsewhere and the 50% inhibition concentration of infections harboring these mutations did not differ from those of isolates without such mutations. Additionally, there was a significant increase in the frequency of isolates with *Pfcrt* K76T alongside increasing in vitro sensitivity of field isolates to CQ, with a reciprocal decline in susceptibility to ART and LM while the response to DHA and PPQ remaining stable during the 14-year study period (2008 and 2021). Studies have shown that parasites with impaired response to antimalarial drugs linger in longer in an infection and are prone to transitioning to gametocytes as the drug dose diminishes [36]. It is therefore noteworthy that the presence of the polymorphisms in *plasmepsin* 2/3 (*Pfpm2/3*) and *Pfmdr1* genes presents a potential risk of rapid selection of resistance in Kenya as drug pressure builds as the use of DHA-PPQ continues to be embraced.

The accuracy of PSA in the determination of PPQ susceptibility makes the method feasible and deployable tool for sustainable surveillance of PPQ resistance. Studies have shown that PSA ≥ 10% corresponds to the PPQ-resistant phenotype [17]. Up to 85% of the isolates tested by PSA had < 10% consistent with sensitivity to PPQ. Our analyses of IC_50_s of those isolates with PSA ≥ 10% versus those with PSA < 10% showed that they were comparable, suggesting that sustained exposure of the parasites to drug in the ex vivo or in vitro assay, for the 72 h could be masking the stage-specific response to PPQ that is well assessed by the PSA.

The putative PPQ resistance polymorphisms in *Plasmepsin*-*2/3* (*Pfpm2/3*) and, *Pfmdr1* genes reported elsewhere were present in Kenyan isolates at low frequencies, suggesting susceptibility to drugs containing PPQ but presenting a potential risk of rapid selection as usage of this regimen becomes widespread in Kenya. Importantly, this study did not find a correlation between these polymorphisms and neither in vitro/ex vivo IC_50_s nor PSA values. However, a significant association between *Pfcrt* 76T SNP, and in vitro PPQ response (IC_50_s) appear to argue for the role of *Pfcrt* SNPs in modulating parasite response to PPQ. The WHO recommends continuous monitoring of the performance of recommended drugs for early detection of resistance [1, 37, 38], and growth inhibition assays and molecular marker analyses are the most scalable and cost-effective drug resistance surveillance methods hence mostly deployed globally [38].

The median piperaquine 50% inhibitory concentration (IC_50_) discerned by this study was 29.40 nM, which is below the resistance cut-off points of 135.00 nM reported in Mali [39] and France [40]. Similarly, median IC_50_s below resistance cut-off have previously been reported in Uganda, Senegal, and Sierra Leone [41–44], suggesting continued sensitivity of parasites from most regions of Africa to PPQ. Additionally, the per-period piperaquine IC_50_ between 2008 and 2021 remained stable below the resistance threshold suggesting sustained parasite susceptibility to PPQ (*P*= 0.1615). This observation was consistent with previous studies across Africa [41, 44]. In 2021, Tumwebaze and coworkers reported increasing PPQ sensitivity in isolates collected from Tororo and Busia Districts in Eastern Uganda [44]. Moreover, studies by Mwai and coworkers analyzing samples collected between 2005 and 2008, as well as Okombo and coworkers analyzing samples spanning from 1995 and 2013 period from the coastal site of Kilifi, reported median IC_50_s of 31.70 nM and 54.00 nM respectively suggesting marginally higher PPQ sensitivity [45, 46]. These marginally higher IC_50_s in the Coastal region of Kenya coincided with a period when ACT use had just been rolled out and could be associated with the presence of residual CQ-resistant parasites that could harbor impaired PPQ response given the inherent molecular similarities between the latter, a bis-quinoline of the former. This could also be evocative of genetic variations in drug resistance molecular markers between *P. falciparum* across transmission regions of Kenya, as previously reported elsewhere during the same period [46]. Continuous parasite susceptibility to PPQ in Kenya could be due to the low PPQ drug pressure, given that it had never been deployed in Kenya and the increasing frequency of chloroquine-sensitive isolates [41]. PPQ parasite response variation between Kisumu East and Malindi study sites could be attributed to the difference in disease burden geographical zones as suggested earlier by a study in Uganda [47].

The development of resistance to a drug has been shown to affect the efficacy of other drugs that share class and targets in the parasite [47, 48]. Expectedly, a significant positive correlation in activities of DHA-ART drug pair was observed, suggesting that treatment failure of DHA will lead to ineffectiveness of ART and vice-versa (cross-resistance); these could be due to the same mechanism of action [47, 48]. This was in line with earlier findings by Briolant and coworkers in a study that showed in vitro response correlation between ART and DHA [49]. There was no significant association between the response of field isolates to piperaquine and the rest of the drugs suggesting that the emergence of resistance to any of these currently used partner drugs and artemisinin derivatives will not affect the efficacy of PPQ. This was in agreement with reports from earlier studies by Muangthin and coworkers [48].

The artemether-lumefantrine ACTs regimen was rolled out as a first-line regimen for the treatment of uncomplicated malaria more than a decade and a half ago in sub-Saharan Africa and has demonstrated good efficacy over time despite reports of artemisinin resistance in SEA and recently in East Africa [11, 12, 49]. However, reports of impaired sensitivity to this drug have been reported in the Cambodia-Thailand border and in Africa, thus raising concerns about the continued use of artemether-lumefantrine (Coartem®) [9, 13, 50, 51]. Chloroquine was previously used as a first-line drug and was withdrawn in 1998 due to widespread resistance. Since its withdrawal, chloroquine-sensitive parasites have emerged in Africa [45]. Our findings on susceptibility analyses of artemether and lumefantrine that are presently in use, in comparison with findings on CQ show a significant; increase in LM and ART alongside a reciprocal decrease in CQ median IC_50_ during the study period stretching between 2008 and 2021_._ These trends suggest decreasing in vitro susceptibility of parasites to LM and ART, although it remains below the WWARN resistance cut-off point of 115.00 nM and 12.00 nM, respectively [39, 40], and increasing parasite’s susceptibility to CQ as reported elsewhere in Africa [44, 52–54]. The observed sensitivity profile trend is due to the withdrawal of CQ drug pressure and increased use of ART and LM in Africa, facilitating selection for parasites resistant to LM, and sensitive to piperaquine, and chloroquine in the region [22, 54, 55]. Consistent with findings from Uganda, no significant change in DHA susceptibility was depicted during the study period [41, 44]. This was in agreement with reports of sustained artemisinin derivatives efficacy in Africa except for Rwanda, suggesting that Kenyan circulating parasite populations are sensitive to artemisinin derivatives [12, 53]. However, recent reports of artemisinin-resistant parasite emergence in Uganda by Balikagala [11] warrant continued surveillance of artemisinin derivatives resistance in sub-Saharan Africa.

Mutations in *Pfpm2/3*, *Pfmdr1*, *Pfdhps*, *Pfdhfr*, and *Pfcrt* have been associated with antimalarial resistance leading to a change of dosing regimen in the Thai-Cambodian border [17–19]. Our assessment of these resistance markers in Kenyan isolates between 2008 and 2021 revealed a low frequency of *Pfpm2/3* and *Pfmdr1* genes copy number variation mutations. This finding agrees with other studies in Africa [35, 55], suggesting that circulating Kenyan parasites are susceptible to PPQ [14, 35, 41, 44, 45]. Importantly, detection of the candidate PPQ resistance markers in Kenya at a lower frequency than SEA emphasizes the need for sustained surveillance as is evocative of rapid selection as PPQ use becomes more widespread.

Multiple copies of *Pfpm3* and *Pfpm2* genes were observed in isolates collected between 2008 and 2021 at 7.1% and 2.1%, respectively. Similarly, *Pfmdr1* amplification was reported in the same period at a prevalence of 3.2%. All these copy number variation amplifications were observed in the endemic lake region, suggesting that resistance to PPQ may emerge from this region once DHA-PPQ use becomes widespread. Despite reports of a low frequency of PPQ resistance markers in the analyzed isolates, our in vitro/ex vivo growth inhibition assays (phenotype) data and findings from other studies in Uganda and Tanzania, piperaquine has demonstrated high efficacy in Africa [35, 44, 45], supporting adoption of DHA-PPQ as an alternative first-line drug for malaria treatment in Kenya and the East African region [56]. Increasing prevalence of *Pfcrt* K76 wild-type allele in Kenyan parasites noted in our study is in line with our earlier findings [29] and other studies and elsewhere in Kenya [34, 52, 53].

The molecular and growth inhibition findings continue to show the return of chloroquine-sensitive, facilitated by the withdrawal of chloroquine and the adoption of AL which selects for K76 allele and lumefantrine tolerance [29]. This study noted the frequencies of *Pfmdr1* N86 allele remained largely unchanged over time which is contrary to what was reported in our earlier study [29], and other studies in Western Kenya [34], Angola, and Uganda [9, 13]. Conversely, our growth inhibition assay findings suggest decreasing the sensitivity of parasites to lumefantrine; hence continuous surveillance of the *Pfmdr1* N86 allele should be done in a large sample size to investigate their role in modulating lumefantrine resistance.

Piperaquine survival assay (PSA), described by Duru and coworkers [17] was successfully adopted by our laboratory and it was used to assess the PPQ susceptibility of field isolates collected from the endemic lake sites of Kisumu, Kenya. This assay has been successfully used for earlier detection of piperaquine resistance in Cambodia [17]. To the best of our knowledge, this is the first report on PSA assay analysis of clinical field isolates from sub-Saharan Africa. We showed that Kenyan field isolates have a median piperaquine survival rate of 0.0% which was below the established PPQ resistance cut-off mark of ≥10% [26, 57]. However, isolates having a PSA rate of ≥10% (PPQ resistance cut-off mark) remained susceptible to PPQ when analyzed using the ex vivo/in vitro malaria SYBR Green I method [31], and did not harbor candidate PPQ-resistant markers in their genome. These findings are contrary to the already reported ones in SEA, a region prone to PPQ-resistant parasites [17, 28], suggesting that parasites from Kenya are sensitive to PPQ.

An association between the PPQ sensitivity and *Pfcrt* single nucleotide polymorphisms in the parasite genome was established by this study. *P. falciparum* transporters; *Pfcrt,* and *Pfmdr1* SNPs have been linked to amino quinolines resistance globally [42], while *Pfdhps* and *Pfdhfr* are associated with sulfadoxine-pyrimethamine resistance [38]. Notably, *Pfcrt* 76T SNP is associated with CQ susceptibility correlated with PPQ sensitivity. *Pfcrt* SNPs were shown to affect parasite response to PPQ, *Pfcrt* K76T mixed species genotype conferred higher median in vitro PPQ IC_50_s than the wild type contrary to earlier findings by Koleala et al. (2015) and Rasmussen et al. (2017) [41, 54]. Moreover, there was no association between PPQ sensitivity and *Pfmdr1* N86, 184F, *Pfdhps,* and *Pfdhfr* polymorphisms that confer resistance to LM, MQ, and SP, respectively, as previously reported in Africa and SEA [26, 38, 42, 58], therefore suggesting PPQ susceptibility in malaria-endemic countries prone to LM, and CQ resistance. Absence of a significant association between PPQ and SP resistance mutations (*Pfdhps* and *Pfdhfr*), suggests that SP resistance parasites are sensitive to PPQ, and this further supports the adoption of DHA-PPQ as the next drug for seasonal malaria chemoprevention in sub-Saharan Africa areas prone to SP resistance [35].

This study is subject to several limitations. First, PPQ has a long half-life and remains at an adequate effective dose as the parasite transitions to multiple stages. PSA assay was not designed to analyze PPQ sensitivity to different parasite stages through drug pulsing; hence it doesn't exhaustively represent the mechanism of drug action and resistance. Secondly, DNA degradation amid sample storage, handling, freezing, and thawing might possibly have biased the tests towards detection of wild type over mutants in case of parasites genome obtained infections with low density of strains that harbor mutations that confer resistance [35]. Lastly, the genotype-phenotype association study was limited by the small sample size of isolates with genotypic data for *Pfcrt*, *Pfdhps*, *Pfdhfr*, and *Pfmdr1* SNPs since the isolates collected between the 2014 and 2017 period had been used up in other studies.

## Conclusions

This study demonstrates that circulating Kenyan *P. falciparum* parasites are sensitive to piperaquine suggesting the benefits of its use as an alternative first-line drug and PSA can be easily optimized for field surveillance. In combination, genomic analyses and growth inhibition assays are applicable and deployable in Kenya for monitoring PPQ sensitivity. Sustained in vitro and genomic surveillance is needed for continued monitoring of changing parasite susceptibility patterns to currently used antimalarials as the Country moves to the elimination phase.

## Supplementary Information

**Additional file 1: Table S1.** Number of field isolates collected from each study site the period spanning between 2008 and 2021. **Table S2.** Temporal trends in in vitro susceptibility of field isolates to selected antimalarial drugs between 2008 and 2021. **Table S3.** Piperaquine median IC_50_ and interquartile range of *P. falciparum* parasites collected from six different geographical zones between 2008 and 2021. **Table S4.** Temporal trends of piperaquine susceptibility across different study sites during the study period. **Table S5.** Summary of *Pfmdr1* and *Pfpm2/3* copy number at various study sites between 2008 and 2021.

**Additional file 2: Fig S1.** Susceptibility of *P. falciparum* field isolates and controls to antimalarial drugs. **Fig S2.** The frequency of *Pfmdr1*, *Pfpm2* and *Pfpm3* copy numbers in infections during the study period.

**Additional file 3:.** Growth inhibition and genomic end point study data.

## Data Availability

The datasets analyzed during the study are provided as an Additional file 3, but for confidentiality purposes, personal identifying information of the patients has been removed. This data can be made available upon request with a substantive reason to the corresponding author.

## Abbreviations

ART: Artemether
CQ: Chloroquine
DHA: Dihydroartemisinin
LM: Lumefantrine
PPQ: Piperaquine
PSA: Piperaquine survival assay
