# Supplementary material for: Impact of parasite genomic dynamics on the sensitivity of Plasmodium falciparum isolates to piperaquine and other antimalarial drugs
Source: BMC Med. 2022 Nov 18;20:448. doi: 10.1186/s12916-022-02652-2 (PMC9673313; doi:10.1186/s12916-022-02652-2)
Supplement: Supplementary file 1 — Additional file 1: Table S1. Number of field isolates collected from each study site the period spanning between 2008 and 2021. Table S2. Temporal trends in in vitro susceptibility of field isolates to selected antimalarial drugs between 2008 and 2021. Table S3. Piperaquine median IC50 and interquartile range of P. falciparum parasites collected from six different geographical zones between 2008 and 2021. Table S4. Temporal trends of piperaquine susceptibility across different study sites during the study period. Table S5. Summary of Pfmdr1 and Pfpm2/3 copy number at various study sites between 2008 and 2021. [file 12916_2022_2652_MOESM1_ESM.docx]

**Additional file 1**

**Table S1: Number of field isolates collected from each study site the period spanning between 2008 and 2021**

|  | **KDH** | **KOM** | **KSI** | **KCH** | **MGT** | **MDH** | **TOTAL** |
| --- | --- | --- | --- | --- | --- | --- | --- |
| **2008-2013** | **11** | **11** | **17** | **11** | **1** | **7** | **58** |
| **2014-2017** | **21** | **37** | **22** | **13** | **12** | **2** | **107** |
| **2018-2021** | **22** | **36** | **8** | **4** | **11** | **6** | **87** |
| **TOTAL** | **54** | **84** | **47** | **28** | **24** | **15** | **252** |

KDH- Kisumu East County hospital, KOM- Kisumu West sub-county hospital, KSI- Kisii County hospital, KCH- Kericho County hospital, MGT- Marigat Sub-county hospital and MDH- Malindi Sub-county hospital.

**Table S2: Temporal trends in in vitro susceptibility of field isolates to selected antimalarial drugs between 2008 and 2021**

| **Study period** | **2008-2013** |  | **2014-2017** |  | **2018-2021** |  |  |
| --- | --- | --- | --- | --- | --- | --- | --- |
| **Drugs** | **Median IC_50_s (IQR)** | **n** | **Median IC_50_s (IQR)** | **n** | **Median IC_50_s (IQR)** | **n** | ***P*-value** |
| Lumefantrine | 26.3 (5.1-64.3) | 46 | 17.2 (4.5-80.4) | 82 | 62.4 (26.9-100.8) | 48 | 0.0201** |
| Chloroquine | 15.3 (7.6-30.4) | 29 | 16.5 (9.3-42.2) | 65 | 10.4 (7.2-20.9) | 51 | 0..0318** |
| Artemether | 2.7 (0.3-4.3) | 42 | 3.7 (2.3-5.7) | 98 | 4.4 (3.0-7.4) | 49 | 0.0021** |
| Piperaquine | 32.7 (20.2-45.6) | 51 | 27.6 (12.5-47.6) | 105 | 27.3 (6.9-52.8) | 83 | 0.1615 |
| Dihydroartemisinin | 2.8 (1.4-11.1) | 31 | 5.2 (2.0-19.0) | 64 | 7.2 (2.5-14.1) | 58 | 0.1265 |

IQR Interquartile range (nM), n sample size. ** represent statistically significant change in IC_50_ with *P < 0.05.*

Significant changes in susceptibility for each drug across the three study periods were calculated by the Kruskal Wallis H-test

**Table S3: Piperaquine median IC_50_ and interquartile range of *P. falciparum* parasites collected from six different geographical zones between 2008 and 2021**

|  | **Kisumu West** | **Kisumu East** | **Kisii** | **Kericho** | **Marigat** | **Malindi** |
| --- | --- | --- | --- | --- | --- | --- |
| **PPQ median IC_50_ (nM)** | 30.8 | 37.2 | 27.6 | 22.4 | 15.1 | 18.1 |
| **Interquartile range (nM)** | 16.2-47.1 | 19.0-53.2 | 12.7-47.2 | 10.8-58.3 | 6.3 -55.5 | 6.2-35.5 |
| **N** | 82 | 52 | 45 | 27 | 24 | 15 |
|  | | | | | | |

***P* value** = 0.0451 **

Significant diffence was observed betweeen Kisumu west and Malindi hospitals (*P* = 0.0451).

** represent statistically significant change in parasite response to PPQ with *P < 0.05* analyzed by the Kruskal-wallis H test. N- Sample size.

**Table S4: Temporal trends of piperaquine susceptibility across different study sites during the study period.**

| **Hospital sites**  **Median IC_50_ (IQR), n** | | | | | | |
| --- | --- | --- | --- | --- | --- | --- |
| **Study period** | | **KDH** | **KOM** | **KSI** | **KCH** | **MDH** |
| **2008-2013** | | **24.6 (15.5-43.0), 9** | **32.6 (20.1-42.2), 11** | **36.5 (30.9-56.0), 14** | **38.3 (20.2-127.3), 5** | **22.3 (7.8-35.5), 7** |
| **2014-2017** | **37.7 (19.3-51.1), 21** | | **32.4 (21.8-50.3), 36** | **23.2 (9.7-29.4), 22** | **18.9 (5.4-35.8), 9** | **8.7 (3.1-14.2), 2** |
| **2018-2021** | **36.7 (22.8-44.1), 9** | | **27.5 (5.9-52.8), 36** | **15.6 (6.6-52.5), 7** | **18.1 (6.6-28.9), 4** | **12.6(4.9-50.8) ,6** |
| ***P value*** | **0.6670** | | **0.3085** | **0.0485**** | **0.1271** | **0.5647** |

KDH- Kisumu East County hospital, KOM- Kisumu west sub-county hospital, KSI- Kisii County hospital, KCH- Kericho County hospital, MGT- Marigat Sub-county hospital and MDH- Malindi Sub-county hospital. ** represent statistically significant change in parasite response to piperaquine *P* < 0.05.

**Table S5: Summary of *Pfmdr1* and *Pfpm2/3* copy number at various study sites between 2008 and 2021**

| **Hospital sites** | | | | | | | |
| --- | --- | --- | --- | --- | --- | --- | --- |
| **Gene** | **Copy number variation** | **KDH** | **KOM** | **KSI** | **KCH** | **MGT** | **MDH** |
| *Pfmdr1* | Single copy | 60 | 61 | 62 | 40 | 23 | 25 |
|  | Multiple copies | 1 | 2 | 1 | 0 | 4 | 1 |
| *Pfpm2* | Single copy | 62 | 61 | 64 | 37 | 30 | 26 |
|  | Multiple copies | 1 | 2 | 0 | 3 | 0 | 0 |
| *Pfpm3* | Single copy | 59 | 57 | 66 | 38 | 30 | 26 |
|  | Multiple copies | 3 | 7 | 3 | 2 | 1 | 4 |

*Pfmdr1; Plasmodium falciparum* multidrug resistant 1 gene, *Pfpm2*; *Plasmodium falciparum* *plasmepsin* 2;

*Pfpm3*; *Plasmodium falciparum* *plasmepsin 3*. KDH- Kisumu East County hospital, KOM- Kisumu West sub-county hospital, KSI- Kisii County hospital, KCH- Kericho County hospital, MGT- Marigat Sub-county hospital and MDH- Malindi Sub-county hospital.
