# Supplementary material for: Impact of parasite genomic dynamics on the sensitivity of Plasmodium falciparum isolates to piperaquine and other antimalarial drugs
Source: BMC Med. 2022 Nov 18;20:448. doi: 10.1186/s12916-022-02652-2 (PMC9673313; doi:10.1186/s12916-022-02652-2)
Supplement: Supplementary file 2 — Additional file 2: Fig S1. Susceptibility of P. falciparum field isolates and controls to antimalarial drugs. Fig S2. The frequency of Pfmdr1, Pfpm2 and Pfpm3 copy numbers in infections during the study period. [file 12916_2022_2652_MOESM2_ESM.docx]

**Additional file 2**

**Figure S1**. **Susceptibility of *P. falciparum* field isolates to antimalarial drugs.** PPQ; Piperaquine, ART; Artemether, DHA; Dihydroartemisinin, LM; Lumefantrine, CQ; Chloroquine. Reference clones D6 and 3D7 (Sensitive to chloroquine), W2 (Resistant to chloroquine)

**
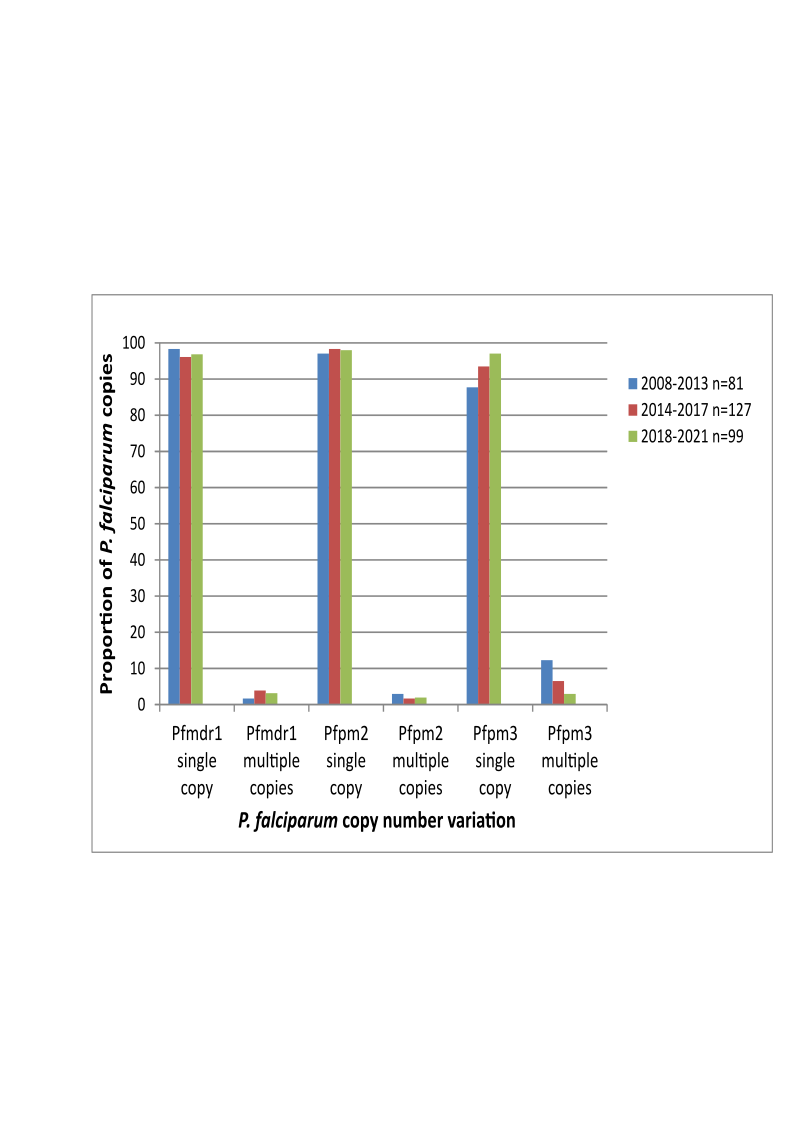
**

**Figure S2**: The frequency of *Pfmdr1*, *Pfpm2* and *Pfpm3* copy numbers in infections during the study period. *Pfmdr1; Plasmodium* *falciparum* multi-drug resistance 1 gene, *Pfpm2*; *Plasmodium falciparum* *plasmepsin*-2 gene, *Pfpm3*; *Plasmodium falciparum plasmepsin-*3 gene
